# Case Report: Adolescent-Onset Isolated Nephronophthisis Caused by a Novel Homozygous Inversin Mutation

**DOI:** 10.3389/fgene.2022.847397

**Published:** 2022-05-18

**Authors:** Zhengxia Zhong, Xiaoyong Yan, Zhengying Fang, Yijun Dong, Jiaxing Tan, Jingyuan Xie, Linhong Hu, Shibin Zhang, Wei Qin

**Affiliations:** ^1^ Division of Nephrology, Department of Medicine, Affiliated Hospital of Zunyi Medical University, Zunyi, China; ^2^ Department of Nephrology, Shanghai Ruijin Hospital, Shanghai Jiao Tong University School of Medicine, Shanghai, China; ^3^ Division of Nephrology, Department of Medicine, West China Hospital, Sichuan University, Chengdu, China; ^4^ Laboratory Animal Centers, Zunyi Medical University, Zunyi, China

**Keywords:** adolescent-onset, homozygous mutation, INVs, NPHP2, ESRD

## Abstract

**Objective:** Nephronophthisis (NPHP) is a rare autosomal recessive inherited kidney disease that can cause cystic enlargement of the kidneys, and lead to end-stage renal disease (ESRD) before the age of 30 years. Herein we describe a case of adolescent-onset NPHP with a novel homozygous mutation in the inversin gene (*INVS*).

**Methods:** The patient was a 15-year-old Chinese boy who presented with ESRD. Genetic testing was performed via whole exome sequencing and validated *via* Sanger sequencing. A novel homozygous *INVS* mutation was identified (c. 1909C > T; p. Gln637Ter).

**Results:** The results of laboratory examinations included urinary protein 1.05 g/24 h, urine erythrocyte count 5/high-power field, serum creatinine 1,026.2 μmol/L, and estimated glomerular filtration rate 5.8 ml/min/1.73 mm^2^. Extrarenal features included hypertension and moderate anemia, and his parents were consanguineous (first cousins). A homozygous 1-bp substitution resulting in a nonsense mutation (c. 1909C > T; p. Gln637Ter) in exon 15 of *INVS* was detected via whole exome sequencing, and validated via Sanger sequencing. According to the classification system of the American College of Medical Genetics and Genomics, the mutated gene in *INVS* is strongly pathogenic (PVS1+PM2+*P*P3+*P*P5). His parents and a younger brother were heterozygous carriers. Based on the above results he was diagnosed with juvenile type 2 NPHP. He underwent hemodialysis, and received a kidney transplant after 2 months. He is currently recovering well, with a serum creatinine level of 117 μmol/L and an estimated glomerular filtration rate of 79.6 ml/min/1.73 mm^2^.

**Conclusion:** Here we have described an extremely rare case of adolescent-onset type 2 NPHP caused by a homozygous *INVS* mutation. The patient had progressed to ESRD by the age of 15 years. The current report will deepen our understanding of the clinical and genetic basis of this disease.

## Introduction

Nephronophthisis (NPHP) is an extremely rare autosomal recessive kidney disease characterized by renal tubulointerstitial lesions, tubular basement membrane disruption, and renal cyst formation that progresses to end-stage renal disease (ESRD) in children (Otto et al., 2003). In addition to renal symptoms NPHP is often complicated with diverse extrarenal features, and approximately 20% of patients suffer from ciliopathy syndrome, including retinal degeneration, bone abnormalities, and liver fibrosis (Srivastava et al., 2017). Genetic studies have identified more than 20 genes that are causally associated with NPHP in humans. Juvenile type I NPHP (NPHP1) is most commonly caused by homozygous pathogenic variants in *NPHP1*, which encodes an SH3 domain protein (Hildebrandt et al., 1997a; Otto et al., 2008). Type 2 NPHP (NPHP2) has also been identified in patients with infantile NPHP, and is distinguished from other types of NPHP by an early age of onset and cystic enlargement of the kidneys. O’Toole et al. (2006) first reported *NPHP2* mutation in a 2-year-old Arab boy with retinitis degeneration and kidney failure. Here in we report the novel *INVS* mutation p. Gln637Ter in an adolescent patient with NPHP2.

## Case

**FIGURE 1:**
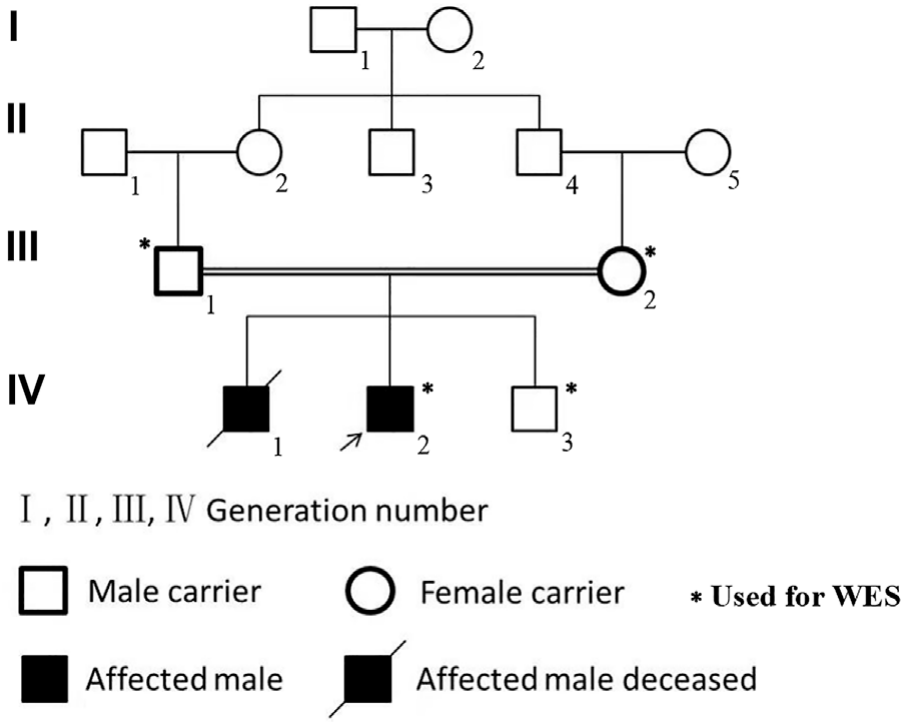
The pedigree of the family with NPHP2/INVS mutation (c.1909C > T) in the exon 15. Affected brothers of INVS mutation are represented by black symbols. Males are represented by squares, and females are represented by round symbols. Crossed symbols represent deceased individuals.

A 15-year-old Chinese boy who had been experiencing headache and vomiting for 1 week was admitted to the hospital (IV-2 in Figure 1). Ophthalmic examination did not reveal any obvious abnormalities, there were no neurological symptoms, and he did not exhibit short stature. His clinical symptoms were sudden and severe, and mainly manifested as hypertension and severe kidney failure. His blood pressure was 170/123 mmHg, his serum creatinine level was 1,026.2 μmol/L, and no prior symptoms were reported. His parents were consanguineous (first cousins), and his older brother had succumbed to an undiagnosed kidney disease at 8 years of age, without a kidney biopsy. The current patient was initially admitted to our medical center on 21 May 2021.

Ultrasonography depicted small kidneys and diffuse lesions (85–90 mm in diameter) in both kidneys, but no cystic enlargement of the kidneys. Laboratory tests revealed a hemoglobin level of 67 g/L and a urinary protein level of 1.05 g/24 h. Chest computed tomography was normal. Laboratory investigations on admission included screening for hepatitis B virus surface antigen, antinuclear antibodies, anti-double-stranded DNA antibodies, anti-glomerular basement membrane antibodies, and anti-neutrophil cytoplasmic antibodies, all of which were negative. The patient was started on hemodialysis. Fortunately he received a kidney transplant after 2 months of dialysis. He is currently recovering well, with a serum creatinine level of 117 μmol/L and an estimated glomerular filtration rate of 79.6 ml/min/1.73 mm^2^. Whole exome sequencing was performed to further investigate the cause of his illness, and segregation was validated via Sanger sequencing in him and his family. Written informed consent was obtained from all family members.

## Methods

Blood samples were collected and DNA was extracted via a DNeasy Blood Kit (Qiagen, catalogue number 69504). DNA of the human exon region was highly enriched via an Agilent SureSelectHuman All ExonV6 Kit, and whole exome sequencing was performed using the Illumina platform. Sanger sequencing was then used to validate the exome sequencing results, and the loci of variation were analyzed in accordance with the standards and guidelines for the interpretation of genetic variants developed by the American College of Medical Genetics and Genomics in 2015 (Richards et al., 2015).

## Results

**FIGURE 2:**
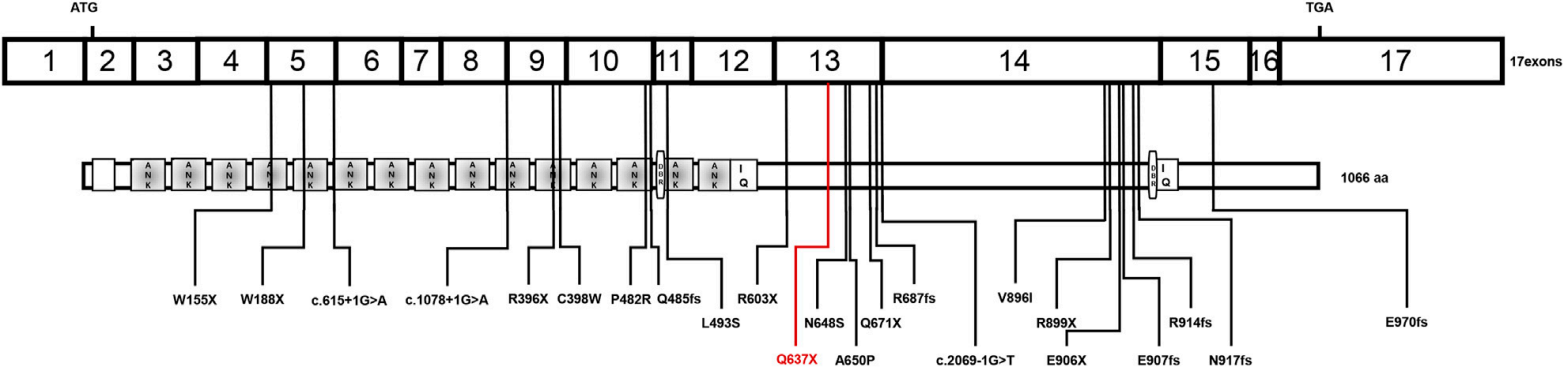
Schematic diagram of the reported INVS mutations. ANK: ankyrin repeats, IQ: IQ calmodulin-binding domains, DBR: destruction box regions.

**FIGURE 3:**
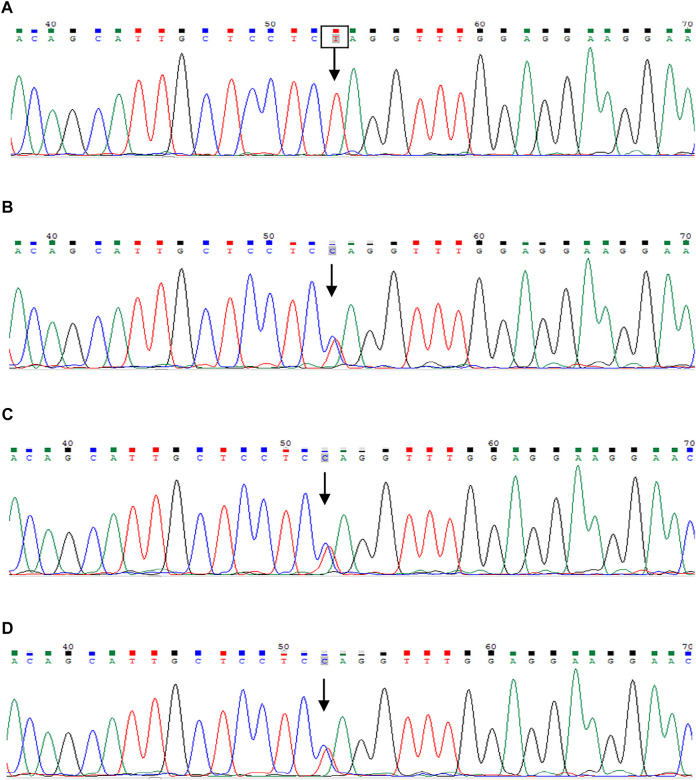
Sanger sequencing of the INVS gene. The patient was identified with 1-bp substitution; **(C)** C1909T, NM_001318382, Chr9:103059299 **(A)**. His parents and younger brother are heterozygous carriers **(B–D)**, and the arrows show the position of the mutation.

Laboratory results included urinary protein 1.05 g/24 h, urine erythrocyte count 5/high-power field, serum creatinine 1,026.2 μmol/L, and an estimated glomerular filtration rate of 5.8 ml/min/1.73 mm^2^. Extrarenal features mainly manifested as hypertension and moderate anemia. His parents (III-1 and III-2 in Figure 1) and younger brother (IV-3 in Figure 1) were in good health, and the results of their medical examinations were normal (Table 1). Mutational analysis of the inversin gene (*INVS*) identified a homozygous mutation in exon 15:c.1909C > T, with a predicted coding sequence change of p. Gln637Ter. A cytosine to thymidine substitution at nucleotide 1909 is predicted to result in glutamine at amino acid 637 being replaced by a stop codon, prematurely terminating the protein (Figure 2). According to the classification system of the American College of Medical Genetics and Genomics the *INVS* mutation identified is strongly pathogenic (PVS1+PM2+*p*P3+*p*P5). The presence of this mutation was then assessed in his immediate family members by sequencing the corresponding site via the Sanger method. The patient’s father, mother, and a younger brother were heterozygous carriers of the same mutation, indicating that it resulted in ESRD in the patient, and originated from his parents who were first cousins (Figure 3). This novel variant has not been reported in the Human Gene Mutation Database or ClinVar before.

**TABLE 1:** Clinical characteristics of the patient and his family.

Parameter	Proband	Father	Mother	Brother
Age (years)	15	50	48	12
Height (cm)	173	165	155	140
Hemoglobin (g/L)	69	151	143	139
Serum albumin (g/L)	42.1	38.9	43.9	47
Scr (μmol/L)	1,026.2	79	69	57
eGFR (mL/min/1.73 m^2)^	5.8	99.5	89.8	148.6
Uric acid (mmol/L)	382	335	207	298
Urine protein (g/24 h)	1.05	0.10	0.09	0.05
U-RBC (/HPF)	5	0	0	0

Scr, serum creatinine; eGFR, estimated glomerular filtration rate; U-RBC/HPF, red blood cell per high power field in urine.

## Discussion

NPHP is an uncommon autosomal recessive renal tubular interstitial disease known to cause renal ciliopathy. It is divided into infantile, juvenile, and adolescent forms based on respective median ages of onset and progression to ESRD of 1, 13, and 19 years. It is reportedly a common genetic condition that leads to ESRD in children and adolescents at the early stage of disease, characterized by polyuria, polydipsia, and anemia (Hildebrandt et al., 2009). Molecular genetic studies have identified more than 20 different *NPHP* genes that cause NPHP (Luo and Tao, 2018; Srivastava et al., 2018). To date the prevalence of NPHP with renal failure has not been thoroughly evaluated. Data from countries other than China indicate an incidence of approximately 1 per 20 million members of the population (König et al., 2017). *NPHP1* causes the most common form, accounting for approximately 20% of cases (Hildebrandt et al., 1997b), but the incidence rate of NPHP in China has not been reported. The clinical features of NPHP can be complex and nonspecific, and they can include growth retardation and anemia followed by progressive deterioration of kidney function. Histological kidney pathology typically includes basement membrane splitting and thickening of renal tubules, and interstitial fibrosis or interstitial cell infiltration.

The current patient was a 15-year-old child who presented with classical symptoms including headache, hypertension, anemia, and renal failure accompanied by ESRD. A novel homozygous pathogenic variant c.1909C > T in exon 15 of *INVS* was detected that is predicted to cause premature truncation of the inversin protein. Ultrasonography of the kidneys revealed hyperechogenic diffuse lesions, and the kidneys were shrunken. Clinically, the opportunity for renal pathological biopsy had been lost, so it was difficult to identify the etiology of his condition. Considering the consanguineous marriage of his parents, and that his older brother had died of ESRD, exome sequencing was performed in his entire immediate family. A novel and strongly pathogenic mutation was identified in the *INVS* gene, which may be associated with the ESRD in this patient. Inversin, the protein defective in NPHP2 patients, localizes to the primary cilia of renal epithelial cells and has been identified in patients with the infantile form of NPHP (Eley et al., 2004). Currently little is known about its specificity and potential interactions. Tory et al. (Tory et al., 2009) described five *NPHP2* mutations that were recurrent in unrelated families, and the frequency of *INVS* mutations is reportedly up to 78% in patients who develop ESRD before 2 years of age. There are previous reports of patients with homozygous *INVS* mutations who developed renal failure before 5 years of age (Otto et al., 2008; O'Toole et al., 2006). Bellavia et al. (Bellavia et al., 2010) also described a patient with an *INVS* mutation who developed ESRD at the age of 11. Previous studies have demonstrated that *NPHP2* mutations can cause ESRD, mainly the infantile-onset NPHP2 form. The age at which previously described patients progressed to ESRD differed from that of the current patient. The novel homozygous gene mutation identified in the current Chinese patient with the adolescent form of NPHP2 has not been previously reported, thus this report adds to the *NPHP2* variation spectrum.

There is currently a lack of effective therapy for NPHP2, so close long-term follow-up and supportive care strategies focused on attenuating the progression of renal impairment are generally utilized, sometimes in conjunction with alternative therapies. Kidney transplantation is preferred over replacement therapy when NPHP2 develops to ESRD. NPHP2 is a rare genetically heterogeneous disease accompanied by insidious clinical features and nonspecific manifestations, and it is easily misdiagnosed. Most patients have progressed to stage 5 chronic kidney disease by the time NPHP is diagnosed, and a kidney biopsy is not feasible due to the small size of the kidneys. It is thus very important to acquire family history, growth history, and other information for the diagnosis of NPHP. The possibility of undescribed pathogenic genes should also be fully considered in younger patients presenting with kidney disease.

The present case demonstrates that the clinical diagnosis of atypical NPHP2 is difficult. We have reported the first severe phenotype in a Chinese NPHP2 patient with adolescent-onset ESRD, and a novel *INVS* mutation was detected via whole exome sequencing. The case serves as a reminder to clinicians that precise medical genetic diagnostics are needed in cases of strongly suspected genetic diseases, especially in patients with a family history of consanguineous marriage.

## Data Availability

The original contributions presented in the study are included in the article/Supplementary Material, further inquiries can be directed to the corresponding authors.
